# Technology platform for facile handling of 3D hydrogel cell culture scaffolds

**DOI:** 10.1038/s41598-023-39081-x

**Published:** 2023-08-07

**Authors:** Hannah Pohlit, Jan Bohlin, Neeraj Katiyar, Jöns Hilborn, Maria Tenje

**Affiliations:** 1 Department of Materials Science and Engineering, Science for Life Laboratory, Uppsala University, Uppsala, Sweden; 2 Department of Chemistry-Ångström, Uppsala University, Uppsala, Sweden

**Keywords:** Biomedical engineering, Biomaterials - cells

## Abstract

Hydrogels are used extensively as cell-culture scaffolds for both 2D and 3D cell cultures due to their biocompatibility and the ease in which their mechanical and biological properties can be tailored to mimic natural tissue. The challenge when working with hydrogel-based scaffolds is in their handling, as hydrogels that mimic e.g. brain tissue, are both fragile and brittle when prepared as thin (sub-mm) membranes. Here, we describe a method for facile handling of thin hydrogel cell culture scaffolds by molding them onto a polycaprolactone (PCL) mesh support attached to a commonly used Transwell set-up in which the original membrane has been removed. In addition to demonstrating the assembly of this set-up, we also show some applications for this type of biological membrane. A polyethylene glycol (PEG)-gelatin hydrogel supports cell adhesion, and the structures can be used for biological barrier models comprising either one or multiple hydrogel layers. Here, we demonstrate the formation of a tight layer of an epithelial cell model comprising MDCK cells cultured over 9 days by following the build-up of the transepithelial electrical resistances. Second, by integrating a pure PEG hydrogel into the PCL mesh, significant swelling is induced, which leads to the formation of a non-adherent biological scaffold with a large curvature that is useful for spheroid formation. In conclusion, we demonstrate the development of a handling platform for hydrogel cell culture scaffolds for easy integration with conventional measurement techniques and miniaturized organs-on-chip systems.

## Introduction

Hydrogels are three-dimensionally cross-linked polymer networks capable of absorbing large amounts of water and are therefore an excellent choice for cell culture scaffolds to mimic the extracellular matrix (ECM). Besides their biocompatibility, hydrogels can be engineered in several ways to match the mechanical stiffness and biological cues present in the native cellular microenvironment^1^. Another advantage of using hydrogels as scaffolds for cell cultures is that their degradability can be tailored, using the cells’ natural capability to degrade ECM components by secreting enzymes to enable cell motility and long-term extracellular matrix remodeling^2^. Hydrogels also provide an opportunity to culture cells in a 3D environment as they would be surrounded by an extracellular matrix in vivo^3^. Thin hydrogel membranes (hydrogel sheets of a few hundred micrometer thickness, in particular, thicknesses below 200 µm^4^) are of specific interest as they show high permeability of nutrients and gasses as well as chemical mediator molecules released by the cells that are necessary for cell–cell communication^5^. Thin hydrogel membranes have been prepared for different applications, e.g. as post-operative anti-adhesive materials^6^ and wet biomaterial sheets^7^, but to make them mechanically robust enough to withstand handling, synthetic materials need to be incorporated, which is not optimal when mimicking in vivo tissue.

A common practice to integrate hydrogel membranes into devices, without the addition of synthetic polymer components, is to prepare them by spin coating or electrospinning, and in a subsequent step freeze-dry and then glue them, to rehydrate upon use^5^. This approach however limits the use of hydrogels to 2D cell culture as cells entrapped in the hydrogel would not survive the freeze-drying procedure. Hence, there is still a need for a facile manipulation method of thin hydrogel membranes, capable of supporting 3D cell cultures. An interesting approach is the dip-casting method as recently presented by the group of Dufva, whereby the scaffold is dipped into a hydrogel precursor solution and the formed hydrogel membrane has a thickness of approx. 600 µm^8,9^. However, in their presented work, the free-hanging films are still rather thick and hence not ideal for 3D cell culture as diffusion alone cannot easily provide the cells throughout the construct with nutrients.

Here, we have used the well-established Transwell platform and modified it to include a thin PCL mesh onto which different types of hydrogels can be molded to thicknesses of 100 µm. Transwell platforms are conventionally used for studying cell barriers by seeding cells on either side of a thin porous polymer membrane. Although very easy to use and an established cell culture analysis platform, the drawback of the Transwells is that they comprise a stiff, synthetic membrane fabricated either in polycarbonate or polyester, which has low in vivo resemblance and does not allow for 3D cultures. Furthermore, transparency of these membranes is very low, and therefore capturing images with microscopy is highly challenging and sometimes impossible. We utilize the advantages of the Transwell setup and adapt it to produce a facile manipulation platform for thin hydrogels. Depending on the chosen application, hydrogels from natural polymers, synthetic polymers, or a mixture thereof can be used. Indeed, it is possible to achieve optically transparent substrates for both cell attachment or inertness, with soft or stiff structures, buckled or flat membranes, as well as being stable or degradable over time^10^. In contrast to conventional Transwell membranes, PCL-supported thin hydrogel membranes can be stacked enabling the combination of different hydrogel materials or hydrogels containing different cell types in one scaffold. In addition, both the hydrogel-liquid interface to the top as well as to the bottom compartment are identical and can be manipulated in stiffness or cell adhesion properties. In previously reported structures where hydrogel is formed on top of the conventional Transwell membrane, this is not the case. Hence, we demonstrate a versatile platform for facile manipulation of different thin hydrogel membranes for cell culture and organs-on-chip applications.

In this study, preparation of the PCL meshes and Transwell scaffold is reported using two different alternatives to fill the PCL meshes with thin hydrogel membranes. Three applications of the scaffolds are presented, including single- or multilayer barrier models and spheroid formation.

## Materials and methods

### PCL thread synthesis

PCL filaments used for mesh winding were produced from granular PCL (DuPont, Capa 6800, MW = 50,000 g/mol) as described previously^11^. Briefly, granular PCL was fed into an extruder as received and extruded to filaments with an average diameter of 0.25 mm. The PCL filaments were wound onto a bobbin, dried under vacuum at room temperature, and stored in a vacuum oven in an aluminum bag until use. The bags consist of commercially available aluminum covered with a polyethene sheet on the inside, often called “vacuum rolls”, which was welded to form bags.

### 3D printing of frames

**Figure 1:**
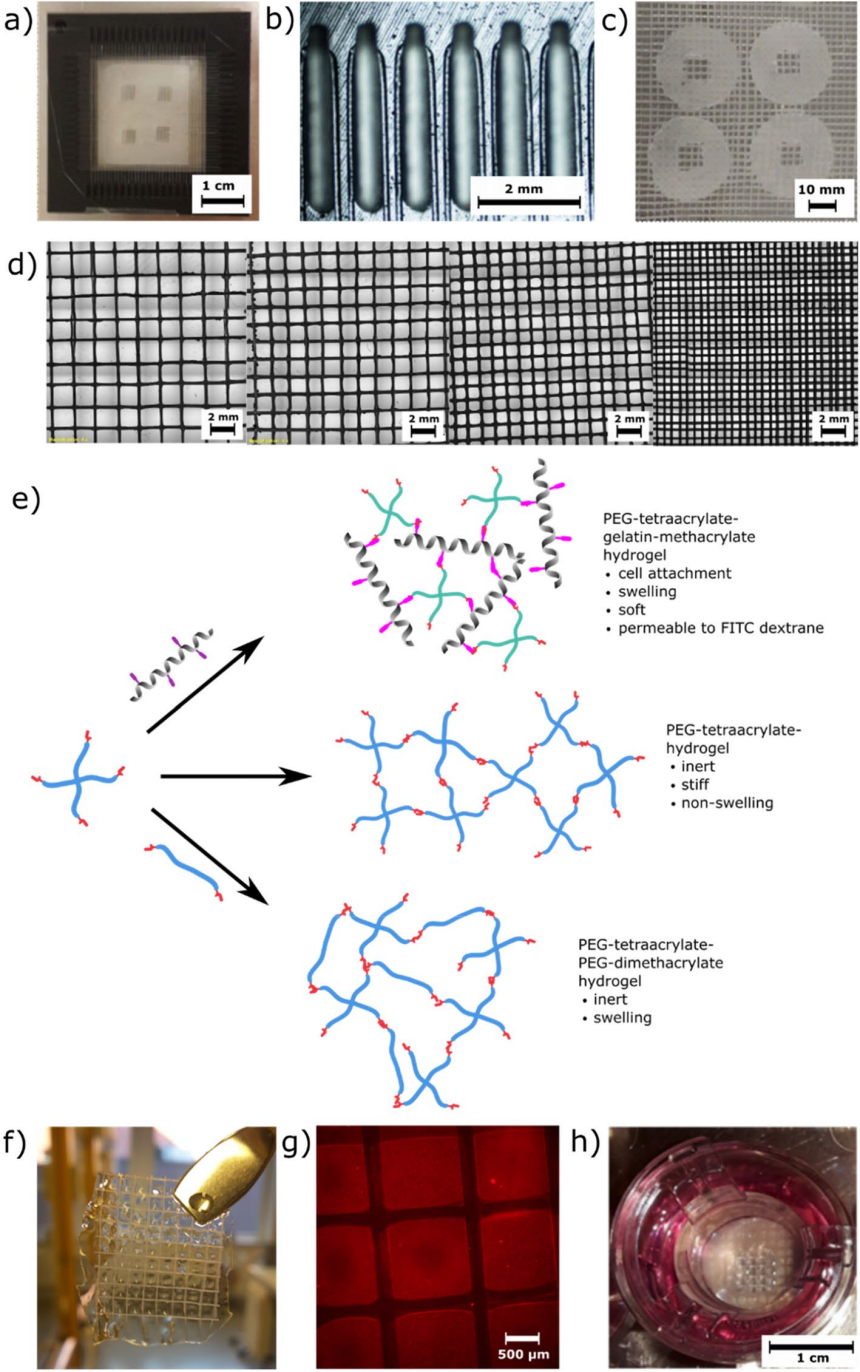
(**a**) 3D-printed frame for PCL mesh formation. (**b**) PCL thread with diameter 100 µm was wound around the pegs of the frame and circular PCL sheets (diameter 12 mm) with an aperture were placed between the threads. Finally, the mesh was formed by “gluing” the threads together with pre-heated copper plates. (**c**) 100 µm-thick PCL supports with mesh pores for the hydrogel with a solid rim. (**d**) Mesh with different pore sizes ranging from 1.8 to 0-4 mm. (**e**) Different hydrogels were used to fill the PCL mesh pores depending on application: PEG-tetraacrylate-gelatin-methacrylate hydrogel, PEG-tetraacrylate hydrogel, or PEG-tetraacrylate-PEG-dimethacrylate hydrogel. (**f**) Mesh pores filled with 100 µm thick non-swelling PEG-4a hydrogel, the mesh support makes it possible to handle the fragile hydrogel membrane with tweezers. (**g**) Fluorescent image of mesh pores filled with hydrogel containing red fluorescent nanoparticles. The darker areas are PCL threads. (**h**) Photograph of the modified Transwell (top view) with the PCL mesh filled with hydrogel immediately after synthesis and having been submerged in cell media.

Frames were fabricated from standard resin (resin black or clear resin, Formlabs) on a FormLabs 2 SLA 3D-printer (Formlabs) based on a design made using AutoDesk Fusion software. The thickness of the pegs and spacing between the pegs was varied between 600 μm and 2 mm, resulting in meshes with pore sizes ranging from 200 µm to 1.8 mm, see Fig. 1d and Fig. S7.

### Mesh preparation

Here, the preparation of a 800 × 800 µm^2^ mesh is described as this size was used for cell studies. PCL threads were taken from the vacuum oven and gently stretched by hand to a diameter of 0.1 mm as determined by a Vernier caliper, before being wound around the pegs on opposing sides of the frame, forming two sets of perpendicular layers of threads, Fig. 1a. A 50 µm-thick PCL sheet was prepared by dissolving 300 mg PCL in 10 mL chloroform. The solution was then poured into a round glass mold (diameter 12 cm) and allowed to dry. The sheet was cut into circular disks with a diameter of 12 mm and a central squared aperture of 3.2 × 3.2 mm^2^ (for 9 800 × 800 µm^2^ pores) or 5.1 × 5.1 mm^2^ (for 16 800 × 800 µm^2^ pores) using a laser cutter, depending on the intended number of pores per scaffold. The circular sheets were positioned between the two layers of perpendicular PCL threads on the frame. The PCL threads were then placed between two polished copper plates that matched the inner space and height of the frame and were pre-heated in an oven at 90 °C. Weights were placed on top of the copper plates, to provide enough force to weld the threads together. The weight of the top copper plates and the additional weight was 4 kg in total. After letting the copper plates cool down to room temperature, the mesh was released by opening the two copper plates with a screwdriver. The meshes could then be released from the copper plates by gently pulling them off. The thickness of the prepared meshes was measured with a thickness gauge (Mikrokator nr 509-4, C. E. Johansson) and ranged between 90 and 115 µm. The PCL mesh supports were cut from the surrounding mesh structure with scissors to obtain structures 12 mm in diameter.

### GelMA synthesis

GelMA synthesis was adapted from Lee et al.^12^ Briefly, 1 g bovine gelatin (Bloom 225, Sigma Aldrich) was dissolved in deionized water [DI water, 4% (wt/vol)] and heated to 37 °C. The pH was adjusted to 9 with NaOH (Sigma Aldrich) prior to and in-between every methacrylic anhydride aliquot addition (1.9 mmol per g gelatin in 10 aliquots, Sigma Aldrich). The pH was then adjusted every 30 min until it remained constant followed by stirring vigorously at 37 °C overnight. The crude product was purified by dialysis (MWCO 12,000–14,000 g/mol, Spectra/Por, Thermo Fisher Scientific) against DI water, which was changed twice a day for 4 days. The purified GelMA was lyophilized and stored at −20 °C until further use, yielding 579 mg (56%). The degree of functionalization was determined by ^1^H NMR and calculated to be 54% of the free amino groups of lysine/hydoxylysine residues in gelatin molecules. All spectra were processed using MestreNova from Mestrelab Research S.L.

### PEG-DMA synthesis

The reaction was performed as previously described by the authors^13^. Briefly, PEG(12,000) (1 g, n = 0.0833 mmol, Sigma Aldrich), 3,5-di-*tert*-butyl-4-hydroxytoluol (BHT, 50 mg, Sigma Aldrich) and CALB (lipase B from Candida Antarctica, 200 mg, Sigma Aldrich) were dissolved in toluene (10 mL, VWR) in a flask and heated to 50 °C. Subsequently, vinyl methacrylate (100 μL, 10 eq., n = 0.833 mmol, m = 93.4 mg, Sigma Aldrich) was added and the reaction was performed at 50 °C for 72 h. The CALB beads were removed by filtration and thereafter the solvent was removed by rotary evaporation. The crude product was purified by twofold precipitation from methanol (Thermo Fisher Scientific) into ice-cold diethyl ether (TCI chemicals). The pure product was dried under vacuum for 48 h and stored at −20 °C until use. The product was analysed with ^1^H NMR, see Fig. S6, yield 0.852 g (85%). NMR spectra were recorded in DMSO-d_6_ on a Bruker 400 MHz spectrometer. The degree of functionalization was determined by ^1^H NMR and calculated to be 98.5% of all hydroxyl end-groups. All spectra were processed using MestreNova from Mestrelab Research S.L.

### Hyaluronic acid-acrylamide synthesis

The reaction was performed as described by Shi et al.^14^ Briefly, sodium hyaluronate (Lifecore Biomedical) reacted with *N*-(2-aminoethyl) acrylamide linker (abcr GmbH) to functionalize the carboxylic acid group of HA with acrylamide groups. After purification, the final product was freeze-dried and stored at − 20 °C until further use. The degree of modification was determined by ^1^H NMR to be 14%.

### Hydrogel precursor solutions

Different compositions of hydrogels were investigated with respect to their “pocket” formation capability, i.e. swelling behavior (for spheroid formation applications) and stiffness (for barrier model applications). Hydrogels comprised 1.5–3 wt% of PEG-tetraacrylate (PEG-4a, Sigma Aldrich) and 1–7.5 wt% of PEG-dimethacrylate (PEG-DMA, Signa Aldrich) or 6–10 wt% GelMA.

The hydrogel precursor solution was prepared by mixing the chosen percentage of polymer, e.g. 1.5% (w/v) PEG(10,000)-tetraacrylate and 7.5% GelMA (w/v) with 0.1% (w/v) lithium phenyl-2,4,6-trimethylbenzoylphosphinate (LAP, Sigma Aldrich) in phosphate-buffered saline (DPBS, Thermo Fisher Scientific, 1:9 diluted with DI water).

### Sol–gel analysis

A sol‐gel analysis was performed to gain insight into the cross‐linked and non‐cross‐linked proportion of the reactants following the procedure described by Nagasawa et al.^15^ After cross-linking, hydrogels were dried in an oven (Hybaid Shake ‘n’ stake, Thermo Fisher Scientific) at 70 °C until the weight remained constant. The gel content of the dried samples was determined by weighing the insoluble part after extraction in PBS for 48 h at room temperature. The gel fraction was calculated with the following formula:

$$Gel\, fraction \,\left(\%\right)\,=\,{W}_{d}{/W}_{i}\,\times \,100$$

where W_i_ is the initial weight of the dried sample after UV cross-linking and W_d_ is the weight of the dried insoluble part after extraction with water.

### Rheology measurements

Rheology measurements were performed on hydrogel disks (8 mm in diameter and 1.5 mm in height) using an 8 mm stainless steel parallel plate geometry on a Discovery Hybrid Rheometer 2 (TA Instruments). Measurements were conducted at an axial force of 30 mN at 37 °C. Frequency sweep experiments (0.1–8 Hz at an oscillation strain of 0.267%) were performed as triplicates, and the storage modulus is reported from an average value at 1 Hz.

### Preparation of suspended hydrogel structures

In this work, we used two different alternatives to connect the suspended hydrogel membrane to the Transwell support structure. In the first approach, the PEG/gelatin hydrogel precursor solution is deposited into the net structure and cured via a short UV exposure before being bonded to the Transwell support (alternative 1). Alternatively, the complete support structure, including the Transwell frame and PCL nets, were first assembled before the mesh pores were filled with hydrogel that was cross-linked (alternative 2).

Alternative 1 (see Fig. S1): glass slides were covered with a stretched layer of parafilm prior to use. The PCL supporting mesh was placed on the glass slide and a drop of 50 µL hydrogel precursor solution was added. The drop was spread out using the pipette tip to ensure all pores were covered, before a second glass slide was placed over the mesh. The glass slides were pressed together during the cross-linking process. The hydrogel precursor solution was cross-linked using a handheld UV-lamp (365 nm, APM UV-Cure, APM-Technica AG) from both sides for 5 s each. The intensity is reported to be 650 mW/cm^2^ at a distance of 2 mm (product manual, AMP-Technica AG). After cross-linking, approx. 500 µL of PBS was injected between the glass slides using a syringe with a flat needle (Hypodermic needle 27G, VWR) to enable the glass slides to be separated without damaging the hydrogel. A drop of PBS (ca. 40 µL) was added on top of the aperture containing the hydrogel, while the attachment rim consisting of sealed PCL pores was dried using fiber-free tissues. The hydrogel-filled PCL support was checked under an inverted microscope (Olympus IX73) equipped with Orca-Flash 4.0 LT digital CMOS camera to exclude broken hydrogel membrane or membranes with air bubbles in the hydrogel.

The membrane was cut out from conventional Transwells (Corning(R) Transwell(R) polyester membrane cell culture inserts, 12 mm, Sigma Aldrich), and the plastic rim was covered with UV glue (Norland Optical Adhesive 81, Norland Products, Inc). The hydrogel-filled PCL mesh support was placed on a clean glass slide and the Transwell positioned on top of it, bringing the UV glue and the attachment rim of the PCL support into contact. While pressing the Transwell down, UV light was used on all sides to cure the glue with a handheld AMP UV-Cure lamp. The Transwell was then immediately placed in cell media (300 µL inside and 1.5 mL outside of the Transwell) to prevent the hydrogel membrane from drying out. The liquid levels should be almost identical to minimize pressure differences affecting the hydrogel membrane, while adding a few additional microliters to the inside compartment ensures all hydrogel pockets to point in the same direction (downwards).

Alternative 2 (see Fig. S1): the equipment used for mesh filling was the same as described in alternative 1. First, the PCL mesh was glued to the Transwell frame using UV glue (described in the section above) and subsequently filled with hydrogel. The scaffold was dipped into a drop of 20 µL hydrogel precursor solution, gently moved on a glass slide to even out the thickness of the hydrogel precursor in the mesh, and finally exposed to UV light. Approx. 500 µL of PBS were added using a syringe with a flat needle to enable, let stand for 5 min and then the Transwell was carefully lifted and transferred into cell media.

### Diffusion studies

For diffusion studies, 3% of red fluorescent polystyrene beads (0.5 µm, Thermo Fisher Scientific) were added to the hydrogel precursor solution to highlight the borders of the hydrogel. Images were acquired using a laser confocal microscope (Leica SP8) equipped with a photon multiplier detector and a hybrid detector. z-stack measurements were performed with a 20× objective to define the hydrogel membrane, together with the liquid compartments above and underneath. 15 z-stacks over a height of 500 µm were measured with the hydrogel membrane in the center. Immediately before the diffusion experiment started, the 300 µL of cell media inside the Transwell scaffold was exchanged for 300 µL of FITC-dextran solution (FITC-dextran of 4, 40, 70, 250 or 500 kDa respectively, at a concentration of 0.15 mg/mL). Diffusion was imaged with z-stacks immediately after FITC-dextran was added, and continued until the fluorescence intensity between the inner and outer compartments reached equilibrium. The z-stacks were then analyzed using a mean intensity quantification tool in the Leica LAS X software. To define the equilibrium of fluorescence intensity, the z-stack closest to the top and another closest to the bottom of the hydrogel membrane were used.

### Hydrogel swelling

**Figure 2:**
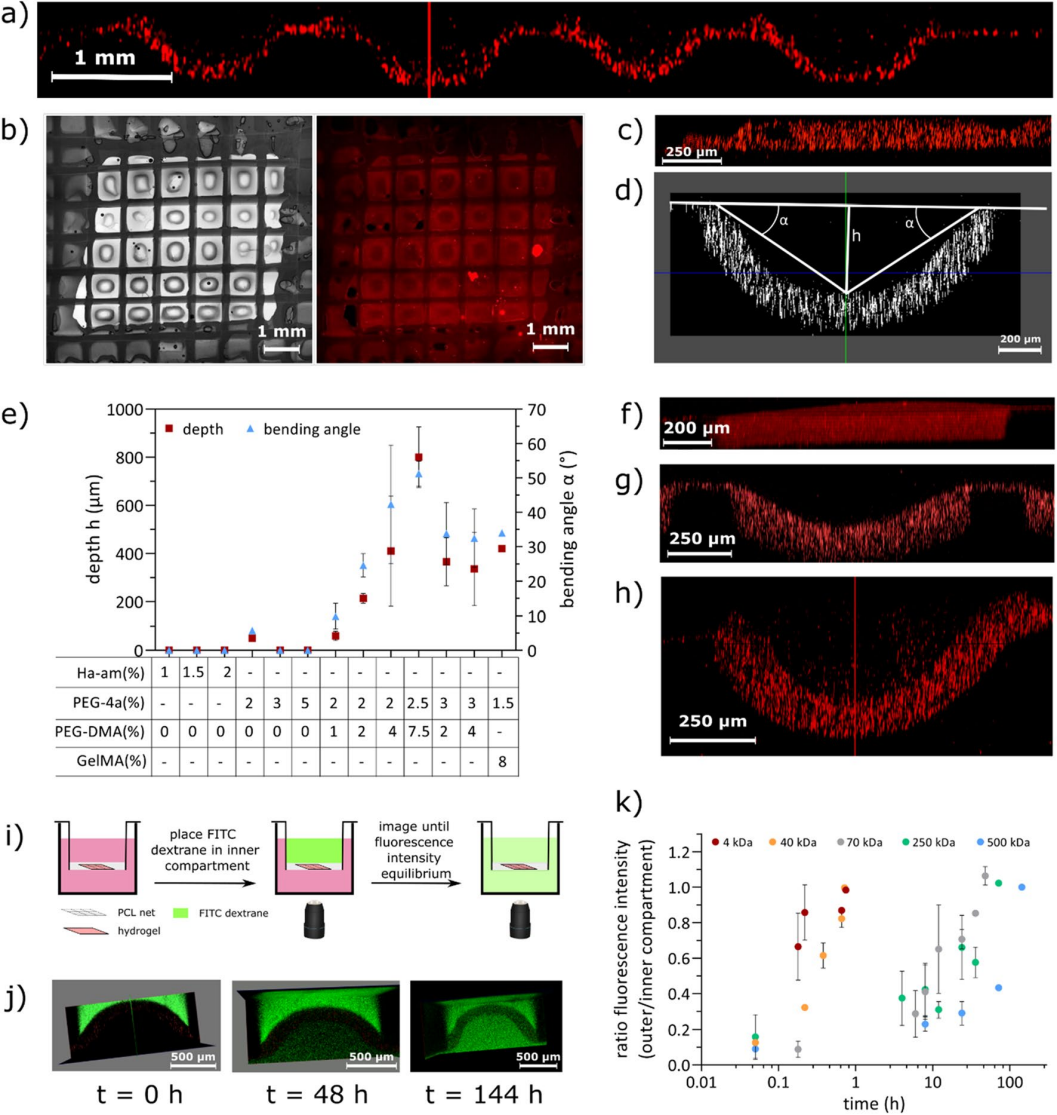
Tunable swelling of hydrogels with or without cell attachment cues. (**a**) Confocal image of several buckled hydrogels composed of 3% PEG-4a + 2% PEG-DMA in side view. (**b**) Confocal image of one scaffold filled with hydrogel composed of 3% PEG-4a + 2% PEG-DMA in top view, all pores buckled. (**c**) Confocal image of one pore filled with 1% hyaluronic acid acrylamide hydrogel in side view. (**d**) Confocal image of one pore filled with 1.5% PEG-4a + 7.5% GelMA. In this picture, the evaluation of the buckling angle (α) and the swelling depth (h) are shown. (**e**) Graph showing the effect of adding linear PEG-DMA to PEG-4a with respect to swelling, pocket depth (red) and buckling angle α (blue) of swollen gels. Hydrogel swelling of PEG-4a + GelMA is displayed as a reference. (**f**) Confocal image of one pore filled with 3% PEG-4a without added PEG-DMA hydrogel in side view. (**g**) Confocal image of one pore filled with 3% PEG-4a + 2% PEG-DMA hydrogel in side view. (**h**) Confocal image of one pore filled with 3% PEG-4a + 4% PEG-DMA hydrogel in side view. (**i**) Schematic of the diffusion study with FITC-dextran of different molecular weights. The hydrogel scaffold filled with PEG/GelMA hydrogel was placed in cell media and subsequently FITC-dextran of differing molecular weights was added to the top compartment. The scaffold was imaged with z-stacks immediately until concentration equilibrium was reached. (**j**) Representative images of the largest FITC-dextran tested, M = 500 kDa over time. Left: hydrogel scaffold directly after FITC-dextran addition. Middle: FITC-dextran diffusion can be seen after 48 h with the naked eye for the highest molecular weight tested. Right: equilibrium is reached after 144 h. (**k**) Diffusion through the hydrogel membrane was monitored by measuring z-stacks with a confocal microscope over time.

Pocket formation of hydrogels composed of 1.5–3 wt% of PEG-tetraacrylate and 1–7.5 wt% of PEG-dimethacrylate or 8 wt% GelMA was investigated by preparing thin hydrogels in the Transwell-mesh support and taking z-stacks through the pores. The deepest point of the hydrogel was located by scrolling though *zx* and *zy* of all z-stack images in 3D mode (see Fig. S5), using the LAS X software for Leica SP8. Subsequently, the buckling angle α and the swelling depth *h* were determined as shown in Fig. 2d. The angles were calculated by positioning a baseline over the hydrogel from the highest point on the left end of the pore to the highest end on the right side of the pore, followed by positioning a perpendicular line at the deepest point of the hydrogel pocket. This second line was used to measure the swelling depth of the hydrogel pocket. Then, the deepest point in the hydrogel pocket was connected to the baseline where the hydrogel starts buckling downwards, and the angle in-between the lines was calculated using InkScape software. Hydrogel buckling was analyzed at least in triplicate per hydrogel composition, with four angles per gel “right” and “left” buckling angle in *zx* and *zy* direction.

### Cell culture

Mouse brain endothelial cells (bEnd.3, ATCC) and Madin-Darby Canine Kidney II cells (MDCK cells, developed and provided from^16^) were cultured in Dulbecco’s Modified Eagle Medium (DMEM) high glucose GlutaMAX (Gibco) supplemented with 10% fetal bovine serum (GE Healthcare HyClone) and 1% Penicillin Streptomycin (Lonza). MC3T3-E1 murine calvarial preosteoblasts (ATCC, CRL-2594) were cultured in Minimum Essential Medium (MEM)-α medium (Gibco), supplemented with 10% fetal bovine serum and 1% penicillin/streptomycin. Human lung fibroblasts (hLF, PELOBiotech GmbH, PB-CH-450-0811) were cultured in Cellovations® Fibroblast Growth Medium (PELOBiotech GmbH, PB-MH-400-9099), supplemented with 2% FCS and 50 ng/ml amphotericin B; 50 ng/ml gentamicin (both included in the medium kit from PELOBiotech GmbH).

The cells were maintained at 37 °C in 5% CO_2_ and cell media was exchanged every second day. Cells were passaged when reaching 60–80% confluency, using TrypLE™ Express enzyme (Gibco) for dissociation. bEnd.3 cells in passage between 24 and 35, MDCK cells in passage 9–12, MC3T3 cells in passage 19–21 and fibroblasts cells in passage 3 were used for experiments. To evaluate barrier formation, the cells were seeded at a density of 30,000 cells/cm^2^ on the hydrogel membrane of the inner compartment and cultured in the respective cell media at 37 °C in 5% CO_2_.

### TEER

The EndOhm chamber (World precision instruments) was cleaned using Tergazyme Enzyme-Active Powdered Detergent diluted 1:100, 70% ethanol, sterile DI water, and cell media prior to use. The media-filled EndOhm chamber was placed in the incubator to equilibrate temperature and CO_2_. The hydrogel scaffold mounted on the modified Transwell cup was placed inside the EndOhm chamber ensuring the same liquid height in the inner and outer compartments to prevent pressure differences affecting the hydrogel membrane. Then, the EndOhm chamber was placed back in the incubator and connected to an impedance analyzer (MFIA impedance analyzer, Zurich Instruments Ltd). After 15 min of equilibration time, TEER measurements were started. TEER values are reported at 1584 Hz.

### Spheroid formation

**Figure 3:**
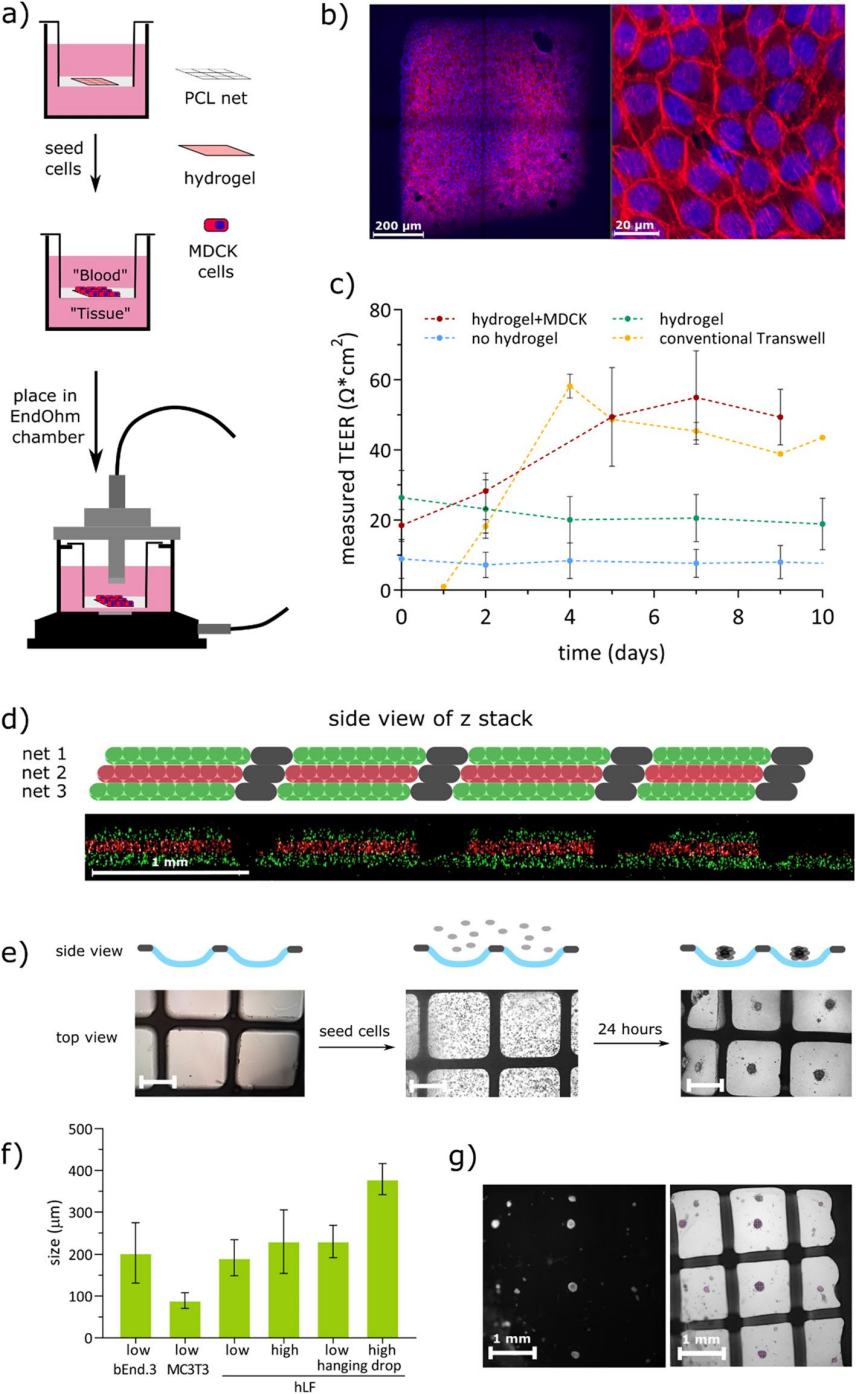
(**a**) Schematic of cell culture on hydrogel scaffold. MDCK formed confluent layers after 7–9 days in culture. TEER measurements to monitor development of the junctions were taken at regular intervals by placing the scaffold inside an EndOhm chamber. (**b**) Cells on the hydrogel were stained with Hoechst 33342 (blue) and phalloidin Alexa Fluor 594 (red). Top view of one pore (left) and zoomed-in view (right). (**c**) Measured TEER from scaffolds without hydrogel (blue), with hydrogel (green), with hydrogel and MDCK cells (red), and MDCK cells on a conventional Transwell membrane (yellow), N = 3 (left). (**d**) Top: schematic representation of the hydrogel-filled stacked meshes. Bottom: confocal image of the PCL mesh, side view of a z-stack showing red or green fluorescent beads trapped in the hydrogel matrix. (**e**) Top: schematic with a side view on the hydrogel pockets formed in the Transwell scaffold. Bottom: microscopy images of the empty hydrogel scaffold, cell seeding and spheroid formation after 24 h. Scale bar = 500 µm. (**f**) Size distribution of formed spheroids from bEnd.3, MC3T3 or human lung fibroblast (hLF) cells under different conditions: low = 5000 cells/hydrogel pocket, high = 10,000 cells/hydrogel pocket and controls with hanging drop method. (**g**) Microscopy images (top view) of the formed spheroids (fluorescence image of cell tracker stained spheroids, left, and bright field image, right). The spheroids were measured from the microscopy images both in x and y direction, number of analyzed spheroids: 4–16 depending on condition.

Fibroblasts, MC3T3 cells or bEnd.3 cells were seeded with a density of either 5000 or 10,000 cells per hydrogel pocket by pipetting the trypsinized cells into the hydrogel scaffold. The scaffolds were placed in the incubator for 24 h. For the hanging drop method, 15 µl of cell suspension was dispensed into a custom-made PDMS droplet holder, and placed inside the lid of a 35 mm tissue culture dish. This further resulted in a spheroid from a hanging drop. Additionally, 2 ml of PBS was added to maintain the moister content of the dish. The spheroids were analyzed by taking microscopy images with an inverted microscope (Olympus IX73) and measuring in the *z* and in *y* directions using ImageJ (Fig. 3f). The number of analyzed spheroids was 4–16 depending on the condition.

### Cell staining

Cells were stained with phalloidin Alexa Fluor 594 and Hoechst 33342, as described previously^17^. Care was taken during washing to avoid breaking the hydrogel membrane. Briefly, the scaffolds were washed three times with transparent MEM (Thermo Fisher Scientific) for 5 min each, and then incubated with 4% paraformaldehyde solution (Biotium) for 10 min at room temperature. The scaffolds were then washed three times with ice-cold PBS for 5 min each. Incubation in 0.05% Triton-X100 (Sigma Aldrich) in PBS for 5 min at room temperature permeabilized the cells. After three more washing steps with PBS for 5 min each, Hoechst (Thermo Fisher Scientific, 1:2000 in PBS) and methanolic phalloidin Alexa Fluor 594 (Thermo Fisher Scientific, 5 U) were added and the scaffolds incubated for 20 min at room temperature. After three additional washing steps with PBS, images were acquired.

### Hydrogel stacking

To prepare the hydrogel precursor solution, 3% (w/v) 4arm-PEG10K-Acrylate (Sigma Aldrich), 0.1% (w/v) lithium phenyl-2,4,6-trimethylbenzoylphosphinate (LAP, Sigma Aldrich), and 1% green or 1% red fluorescent polystyrene beads (10 µm, Thermo Fisher Scientific) were diluted in phosphate-buffered saline (1× DPBS, Thermo Fisher Scientific). The pores of the PCL mesh were filled with hydrogel precursor solution and cross-linked as described above. The meshes were stacked, aligned by eye, and observed between two glass slides on the microscope.

### Software

To create figures (TOC, Figs. 1, 2 and 3, Figs. S1 and S2), Inkscape version 1.2.2 (b0a8486541, 2022-12-01), https://inkscape.org/ was used. Data was analyzed using Microsoft Excel 2019. Data were plotted using GraphPad Prism version 9.0.0 (121) for Windows, GraphPad Software, San Diego, California USA, https://www.graphpad.com (Figs. 2 and 3). Confocal images were analyzed using LAS X software version 3.7.4.23463 for Leica SP8, leica-microsystems.com (Figs. 1, 2 and 3). ^1^H NMR was analyzed using the software Mnova version 14.0.1 from MestReLab Research, https://mestrelab.com/download/mnova/ (Fig. S6).

## Results and discussion

### Scaffold preparation

The cell culture scaffold comprises two parts, a PCL mesh and a PEG/gelatin composite hydrogel filler. The scaffolds were assembled by first preparing the PCL mesh, gluing it to the frame of a conventional Transwell and filling it with hydrogel. Thereafter, the PEG/gelatin hydrogel precursor solution is deposited into the net and cured via a short UV exposure. Alternatively, the mesh pores can first be filled with hydrogel, after which the hydrogel-filled PCL support can be glued to the Transwell frame. Once the scaffold is prepared, cells are seeded onto the scaffold. The whole process is shown schematically in Figure S1 and described in detail below.

### Mesh fabrication

PCL meshes were prepared as mechanical support for the hydrogels and to facilitate hydrogel integration into the Transwell setup or microfluidic devices. The final mesh structure consisted of a solid sheet of PCL that was melted together with a threaded layer into which the PEG/gelatin hydrogel could be integrated. The first step of mesh fabrication was to form the porous layer. PCL threads with a diameter of 100 µm were wounded around the pegs of a 3D printed frame (Fig. 1). Spacing between the pegs was varied between 600 μm and 2 mm, resulting in meshes with pore sizes from 200 µm to 1.8 mm, Fig. 1d. After winding, solid 50 µm thin PCL sheets with an aperture of 3.2 × 3.2 mm^2^ were inserted between the two perpendicular layers of threads before the threads were welded together between pre-heated copper plates to form a mesh support structure of 100 µm total thickness. A pore size of 800 µm was found to be most suitable as a compromise between low hydrogel buckling as a result of swelling, and large cell culture area, and was hence selected for use in all further experiments. For these nets, pore size was measured to be 790 ± 38 µm × 774 ± 48 µm (N = 38) and net thickness of 102.5 ± 5.9 µm (N = 14).

### Hydrogel composition

The PCL mesh can be filled with different hydrogels depending on the application. Here, we used either a tailor-made hydrogel consisting of PEG to give mechanical stability and gelatin methacrylate (GelMA) to enable integrin-mediated cell adhesion to ensure biocompatibility^18^, or pure PEG gels for applications where low cell adhesion would be required.

In the first application, commercially available PEG-tetraacrylate was used together with gelatin methacrylate according to an established procedure^19^. Lithium phenyl-2,4,6-trimethylbenzoyl-phosphinate (LAP) was used as a photo-initiator for light-induced cross-linking reaction, due to its proven low cytotoxicity^20^. UV light was employed to cross-link the hydrogel precursor solution. The optimal hydrogel composition with regards to stiffness and hydrogel handling was found to be 1.5% PEG-tetraarcrylate, 7.5% GelMA and 0.1% LAP in PBS. This was determined by performing cell studies analyzing cell attachment and cell proliferation as well as rheological studies investigating hydrogel stiffness. The chosen composition yielded a storage modulus of 2684 ± 724 Pa after incubation in cell media at 37 °C for 20 h (data not shown). The stiffness of brain tissue is reported to be 685–1875 Pa^21,22^, so the hydrogel composition is a compromise between being as soft as possible whilst also enabling cell growth and good stability of the hydrogel membranes. For spheroid formation, a hydrogel comprising only PEG was chosen as it is important that the supporting structure does not permit cell adhesion. The hydrogel composition was chosen to be 3wt% PEG-(10,000)tetraacrylate and 2wt% PEG(12,000)-dimethacrylate. Hydrogels from pure PEG-tetraacrylate or pure hyaluronic acid-acrylamide were prepared as controls.

The gel content was determined to be 95.7% for hydrogels composed of 3wt% PEG-(10,000)tetraacrylate, 2wt% PEG(12,000)-dimethacrylate and 0.1% LAP and 99.6% for hydrogels composed of 1.5% PEG-tetraarcrylate, 7.5% GelMA and 0.1% LAP.

The hydrogels presented in this manuscript are naturally transparent due to their composition and thereof enable higher transmission for optical experiments and visualization than conventional Transwell membranes. The PCL net structure is non-transparent but cells can be easily examined in the transparent areas where only hydrogel is present. Due to the regularity of the non-transparent PCL net structure, future improvements could enable automatic recognition and focusing on the net structure to enable automatic image acquisition of cells.

### Scaffold assembly

To ensure the hydrogel culture scaffolds were able to be handled with ease, they were mounted onto conventional Transwells by first removing the initially present track-etched polycarbonate membrane with a scalpel. UV glue was added to the Transwell frame which was then mounted on the solid rim of the PCL support and exposed to UV light. The hydrogel can be integrated with the PCL support either before mounting the mesh on the Transwell carrier (Alternative 1) or after (Alternative 2). Thin hydrogels without integrated PCL support cannot be attached to the Transwell after manufacturing following “Preparation of suspended hydrogel structures alternative 1”. When following alternative manufacturing route 2, the hydrogel thickness is not defined and surface tension of the hydrogel precursor solution is not sufficient to form a stable thin membrane over the area of the Transwell (12 mm).

Alternative 1: The hydrogel precursor solution was pipetted into the pores of the PCL mesh, which was then placed between two glass slides and exposed to UV light. Hydrogel thickness was determined by the mesh thickness as it acts to maintain the distance between the two glass slides. After carefully separating the slides, the hydrogel-filled PCL support was immediately attached to the Transwell frame and placed in cell media to prevent it from drying out. This method resulted in 100–250 µm-thin hydrogel membranes (depending on swelling) inside the PCL mesh pores (Fig. 1).

Alternative 2: The PCL mesh can be glued to the Transwell frame and subsequently filled with hydrogel by simply dipping the scaffold into a drop of 20 µL hydrogel precursor solution, gently moving it on a glass slide to even out the thickness of the hydrogel precursor in the pores, and exposing the composite to UV light. This method resulted in 100–450 µm-thick hydrogel membranes (depending on swelling) inside the PCL mesh pores after cross-linking.

### Hydrogel swelling

Hydrogel swelling and the resulting buckling effect on the hydrogel membrane can be tuned by tailoring the hydrogel composition. As the hydrogel is trapped in the pores and unable to expand sidewise (horizontal), it must expand in the vertical direction resulting in “pocket formation” (Fig. 2). The more the hydrogel swells, the more uncontrolled the pocket formation becomes, resulting in heterogeneous/crumpled pockets in the PCL mesh pores. Hydrogels made of pure PEG-tetraacrylate (PEG-4a) or pure hyaluronic acid acrylamide (HA-am) did not show any significant swelling (Fig. 2c, f). By blending linear PEG-dimethacrylate (PEG-DMA) with PEG-4a, an increased swelling was observed due to the disturbance of the otherwise near total cross-linking homogeneity^23^. Swelling of the gel was increased by both decreasing the percentage of PEG-4a and increasing the percentage of PEG-DMA, although the greatest effect was seen for increased concentrations of PEG-DMA. The same was true for 7.5% of methacrylated gelatin (GelMA) when added to the original PEG-4a hydrogel precursor solution (Fig. 2e).

### Hydrogel porosity

To evaluate the porosity of the prepared cell culture scaffolds, a permeability assay using FITC-labeled dextran was performed. FITC-dextran of different molecular weights (4–500 kDa) was diluted in DI water and the fluorescent intensity was followed over time as the molecules diffused across the gel. For imaging, a confocal microscope (Leica SP8) with a 10× objective measuring z-stacks was used. FITC-dextran solution was placed in the top compartment and diffusion was imaged with z-stacks from t = 0 until equilibrium of fluorescence intensity was reached between the two compartments (Fig. 2).

Figure 2k summarizes the different molecular weights of FITC-dextran tested for diffusion. As expected, lower molecular weight molecules could pass the hydrogel membrane faster than higher molecular weight dextran. The smallest molecule tested, FITC-dextran 4 kDa, passed through the membrane in less than one hour, The diffusion rate decreased with increased molecular weight. In Fig. 2j, the diffusion of the largest tested FITC-dextran of 500 kDa is shown, as an example. After 144 h, fluorescence intensity equilibrium is reached. These results demonstrate that the hydrogel membrane itself does not act as a barrier for water-soluble molecules up to 500 kDa (molecular radius of 14.4 nm)^24^ and should enable large molecules like insulin, leptin, transferrin or monoclonal antibodies^25^ to pass through the hydrogel.

### Barrier model

In this manuscript, we demonstrate three different applications of these hydrogel cell culture scaffolds to show proof-of-versatility. A common approach in in vitro modelling is to culture cells to study transport across protective biological barriers^26^, for example to evaluate drug delivery across the skin^27^, intestinal wall^28^, or blood–brain barrier^29^. To demonstrate their application in barrier analysis, the PCL pores were filled with a hydrogel composed of a mixture of synthetic and natural polymers (PEG-4a and GelMA) to support cell adhesion and seeded with kidney cells. MDCK II cells were seeded on the hydrogel and cultured for 10 days. A confluent layer was formed after ~ 7 days, after which the cells were stained with phalloidin Alexa Fluor 594 and Hoechst 33342 for fluorescent imaging, Fig. 3. Important to note is that the synthetic support mesh is incorporated inside the thin, free-hanging hydrogel structure which means that the cultured cells only have limited interaction with this material.

A common method to evaluate barrier formation is to measure TEER across the cellular barrier and follow an increase in TEER values as the cells proliferate and form a tight mono-layer^30^. Such measurements typically require the repeated movement of the cell culture scaffold, which increases the risk of the thin hydrogel membranes breaking as they are transferred from the culture plate into the EndOhm chamber and back onto the culture plate. The PCL support scaffolds prepared here make handling easier for the operator and gentler on the culture scaffold. Here, an EndOhm chamber was used for up to 28 days of measurements.

Transwell supports with a PCL mesh attached but without a hydrogel unsurprisingly showed low TEER values that did not increase over time. Transwell supports with a PCL mesh and hydrogel-filled pores showed a slightly higher TEER value and also demonstrated a small decrease over time, probably due to the hydrolysis of the aliphatic esters generated upon cross-linking of GelMA. The background value was taken from TEER of the hydrogel-filled PCL scaffold, and subtracted from the measured values of the cell-populated scaffolds. Finally, PCL scaffolds with hydrogels where MDCK cells were cultured, showed an increase in TEER over time, until confluency was reached at day 7, see Fig. 3b, c. After day 7, the TEER value reached a plateau and stayed almost constant for up to one month (data not shown). A positive control, in this case a Transwell cup with a solid PCL sheet reported a TEER value higher than 2500 on day 0 that remained constant throughout (data not shown). As additional control, MDCK cells seeded on a conventional Transwell membrane resulted in similar TEER values.

### Multi-layered barrier model

In more complex barrier models, it should be possible to integrate more than one cell type and even cells that require a 3D culture environment^31^. This technique may be beneficial when e.g. engineering a blood–brain barrier model where different cell types need to be correctly positioned. Furthermore, it can provide a multi-layered structure comprising different hydrogels, so that each cell type may be cultured in optimal gel conditions, related to chemical composition and mechanical properties^2^. Only a few approaches have been studied using thin hydrogels, and those demonstrate large height-to-width ratios^32^. No reports on hydrogel stacking are available with free-hanging hydrogel membranes in close proximity.

Here we present a proof of principle study to demonstrate multilayer assembly of hydrogels. Using different PCL meshes loaded with hydrogels. These include either red or green fluorescent beads stacked between two glass slides and imaged using confocal microscopy. The close proximity of the different cell layers is visible in the side view, Fig. 3g.

### Spheroid formation platform

Another potential application of these hydrogel scaffolds is the formation of spheroids, which are spherical multicellular units that has been used in e.g. in vitro tumor models. Conventionally, spheroids are formed by seeding cells in plates that have wells with round, low adhesion bottoms^33^ or in systems using the hanging-drop approach^34^. For increased throughput, a high-density microwell such as Aggrewell™, which is mostly used for embryoid body formation, has also been used for spheroids^35^. These methods are user-friendly but suffer from being made of stiff and impermeable materials. The advantage of using our hydrogel scaffolds is that they are permeable to liquids and small molecules, as well as being more in vivo like with respect to their mechanical properties, thus making it possible to provide the cells with nutrients through the hydrogel support in a biomimetic setting. In addition, hydrogel stiffness and “pocket” size can be readily tailored according to need.

To form spheroids, a low cell-adhesion hydrogel with a large degree of swelling was chosen, i.e. 3 wt% PEG-(10,000)tetraacrylate and 2 wt% PEG(12,000)-dimethacrylate. The swelling properties were optimized to provide stable and distinct “pockets” into which the sedded cells could self-assemble. To ensure that all pockets deflected in the same direction (downwards), the liquid level of the top compartment was set slightly higher than in the bottom compartment after the UV curing step. Cells were seeded on the hydrogel-filled scaffold with a density of 5000 and 10,000 cells per hydrogel pocket, respectively. The formation of spheroids was observed after 24 h, and imaged with either brightfield microscopy or fluorescent microscopy, Fig. 3d.

Here we demonstrated that it is possible to form spheroids in this platform with different cell types, including endothelial cells (bEnd.3), human lung fibroblasts, and bone cells (MC3T3) (Fig. S3). The spheroids measured 203 ± 72 µm in diameter when formed from bEnd.3 (N = 9 spheroids), 191 ± 42 µm for fibroblasts (N = 7 spheroids) and 89 ± 42 µm for MC3T3 (N = 12 spheroids) resulting from the same seeding density of 5000 cells/hydrogel pocket. For a higher cell seeding density, a slight increase in spheroid size was measured, although not statistically significant (230 ± 76 µm for a seeding density of 10,000 fibroblasts/hydrogel pocket, N = 16 spheroids). As comparison, human lung fibroblast spheroids were formed using the standard hanging drop method resulting in a spheroid size of 379 ± 36 µm for high seeding density and 230 ± 37 µmm for low seeding density. The slightly smaller spheroid sizes generated using the membrane is probably due to the cells adapting to a smaller physical space. Results are summarized in Fig. 3.

A uniform and reproducible spheroid formation yield of around 25% was observed. In several of the other pores, multiple spheroids were formed, most likely due to imperfect buckling of the gel, so that several smaller recesses in each PCL pore appeared, Fig. S4.

## Conclusions

In this paper we present a method to integrate thin hydrogel membranes with a PCL mesh to provide an easy and gentle method of handling thin hydrogel membranes for in vitro cell cultures. Hydrogel-based cell culture scaffolds offer high in vivo resemblance, but it is difficult to integrate them into devices or use them for extended culture periods as they are both soft and brittle and therefore easily break during handling. Here, we demonstrate a new hydrogel handling method for in vitro cell models which opens up new possibilities in organ-on-chip applications and supporting industrial translation.

PCL threads can be used to prepare 100 µm thick meshes with different pore sizes. The PCL meshes are attached to a conventional Transwell insert either before or after filling the pores of the mesh with hydrogel. The hydrogel can be tailored according to the intended application to either support or prevent cell attachment. Furthermore, depending on the hydrogel composition, hydrogel swelling and subsequent hydrogel pocket formation can be controlled. We show three different possible applications for the versatile Transwell device. First, thin hydrogel membranes that can be used as cell culture scaffolds to form biological barriers are shown. Here, the hydrogel itself supports diffusion of FITC-dextran, with MDCK cells grown in a confluent layer on the hydrogel. The hydrogel-free scaffold resulted in constant, low TEER values whereas the scaffold with integrated hydrogel showed elevated TEER values that decreased over time. The scaffold with hydrogel and MDCK cells showed an increase in TEER until a confluent cell layer was formed after approx. 7 days. A major advantage of integrating the hydrogel scaffolds with the PCL mesh is that it provides facile handling of the soft cell culture membranes. Second, we show that it is possible to combine several PCL meshes filled with hydrogel to form a layered structure of hydrogels with different compositions. Finally, we demonstrate that the free-hanging hydrogel membranes can be used as a novel way to prepare spheroids from bEnd.3, MC3T3 or fibroblast cells. Key to achieve the 3D assembly of the cells is to prepare a hydrogel membrane that is inert to cell attachment and forms swollen hydrogel pockets upon gel cross-linking. We believe the technology presented in this study opens up for more microfluidic-based in vitro applications of hydrogel cell culture scaffolds that are today limited by the difficult handling of these fragile cell culture scaffolds.

### Supplementary Information

Supplementary Figures.

## Data Availability

The datasets generated and analyzed during the current study are available from the corresponding author on reasonable request.
