# Supplementary material for: Technology platform for facile handling of 3D hydrogel cell culture scaffolds
Source: Sci Rep. 2023 Aug 7;13:12829. doi: 10.1038/s41598-023-39081-x (PMC10406881; doi:10.1038/s41598-023-39081-x)
Supplement: Supplementary file 1 — Supplementary Figures. [file 41598_2023_39081_MOESM1_ESM.docx]

**Supporting Information**

**Technology platform for facile handling of 3D hydrogel cell culture scaffolds**

Hannah Pohlit^1^, Jan Bohlin^2^, Neeraj Katiyar^1^, Jöns Hilborn^2^ and Maria Tenje^1,*^

^1^Department of Materials Science and Engineering, Science for Life Laboratory, Uppsala University, Uppsala, Sweden

^2^Department of Chemistry – Ångström, Uppsala University, Uppsala, Sweden

*Corresponding author email: maria.tenje@angstrom.uu.se


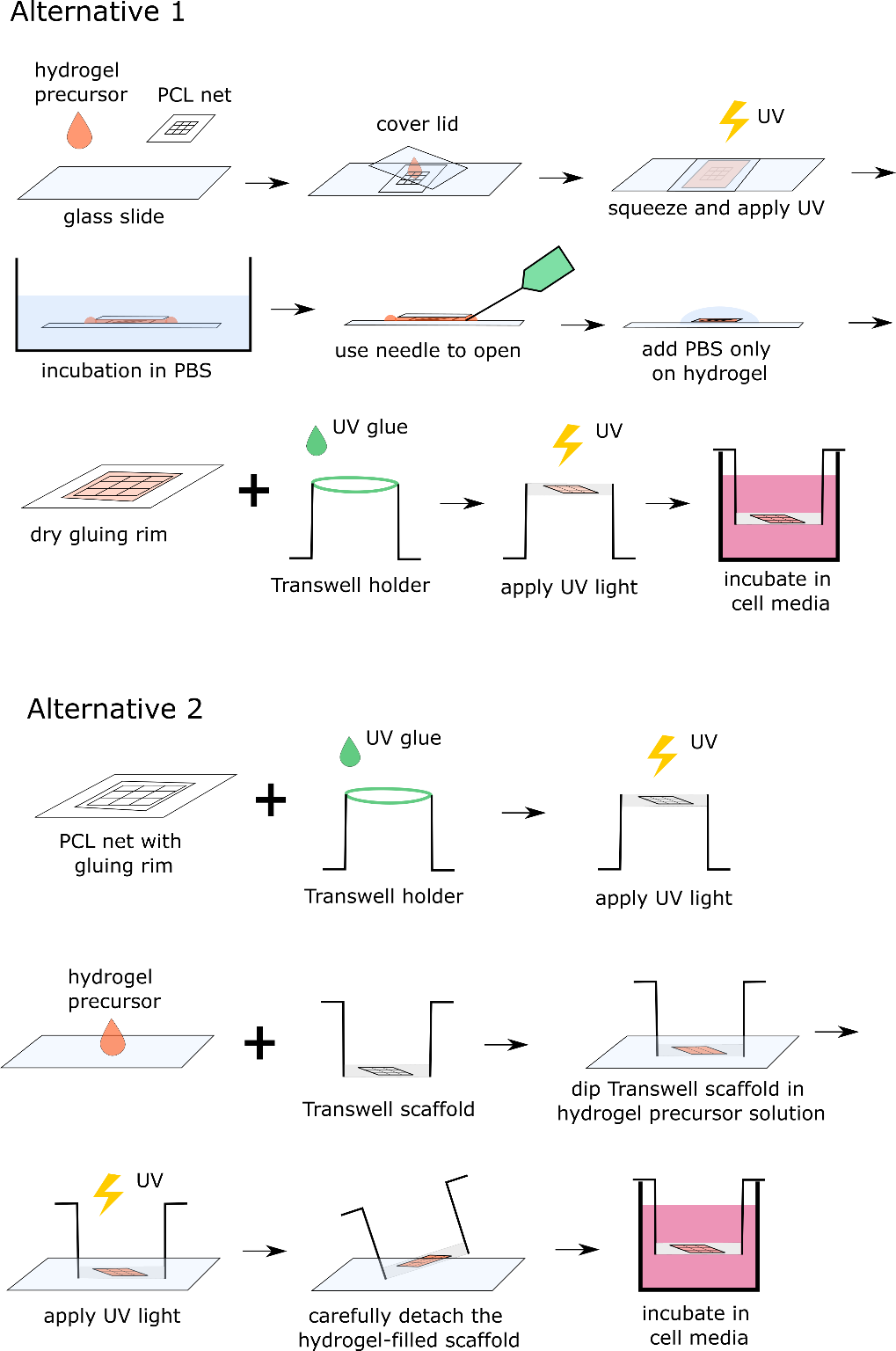


Figure S1: Schematic about the Transwell scaffold assembly process. In alternative 1, the PCL mesh is in a first step filled with hydrogel precursor solution, the hydrogel is cured with UV light when squeezed in between two glass slides and subsequently glued to the Transwell holder using UV glue. In alternative 2, the first step is to assemble the Transwell scaffold using UV glue and filling the mesh pores with hydrogel subsequently. The assembled Transwell scaffold is dipped in a drop of precursor solution and gently moved on the glass slide surface to evenly distribute the liquid. Curing the hydrogel with UV light gives the final scaffold. The assembled Transwell scaffold is immediately placed in cell media to prevent drying of the hydrogel.


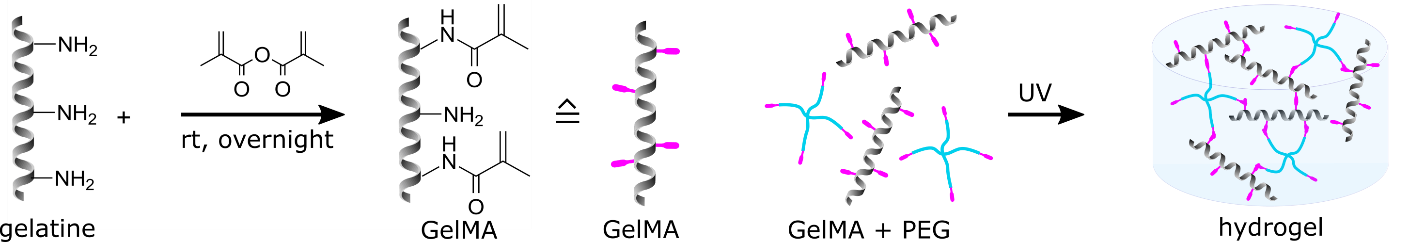


Figure S2. Synthesis scheme of methacrylated gelatine, GelMA. Gelatine is reacted with methacrylic anhydride to form GelMA. GelMA and 4-arm PEG-acrylate are mixed and crosslinked with UV light to form a hydrogel.


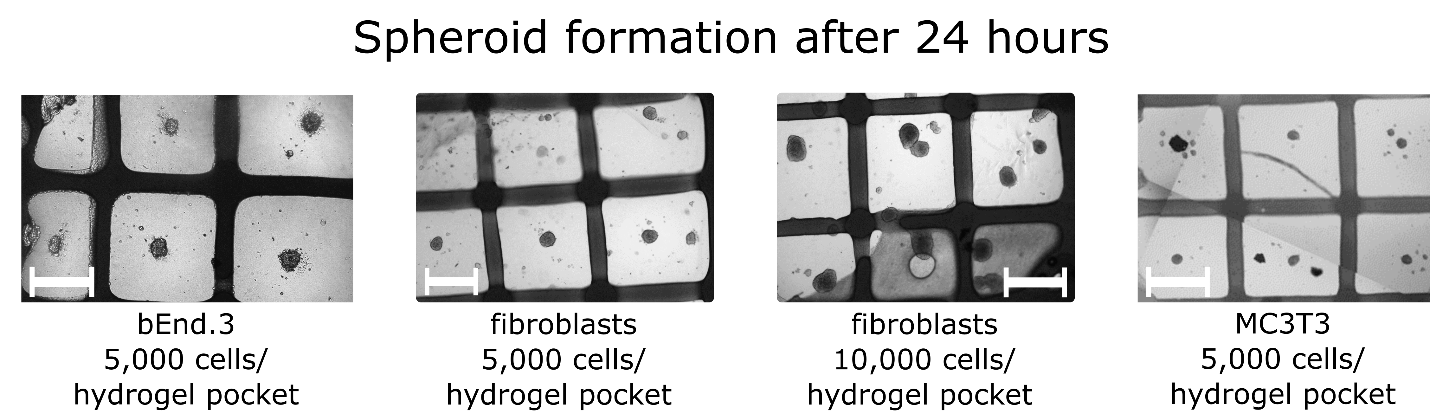


Figure S3. Spheroid formation from different cell lines after 24 hours. Spheroids were formed from bEnd.3 cells, human fibroblasts and MC3T3 cells. Scale bar = 500 µm.


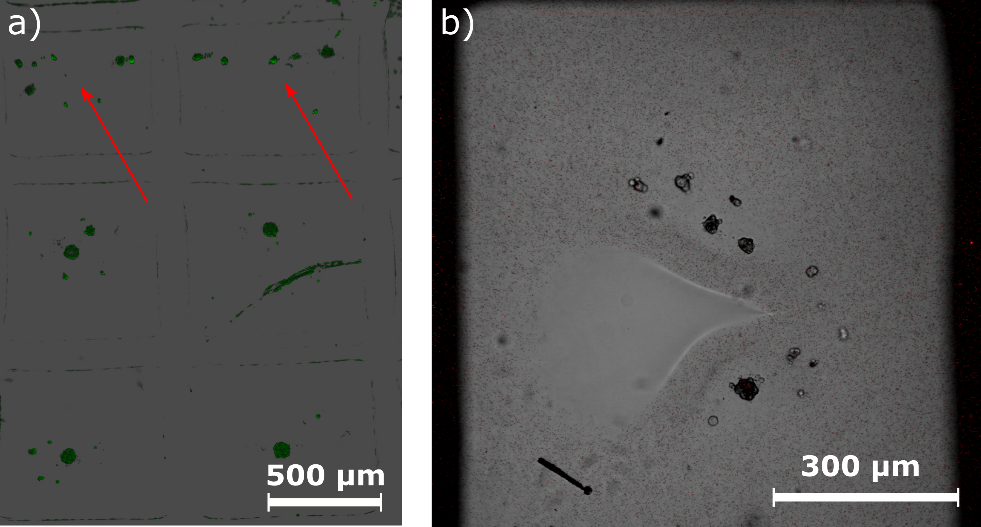


Figure S4. Micrographs of spheroids formed in 6 hydrogel pockets. In the top two hydrogel pockets, several spheroids were formed as the hydrogel did not have a uniformly formed buckled shape but instead had formed an elongated crater, so the cells collect in several smaller pockets and form multiple spheroids. a) Fluorescent image of MC3T3 spheroids stained with cell tracker. b) Bright-field microscopy image of several MC3T3 spheroids formed in an uneven swollen hydrogel pocket. The hydrogel crater is visible due to fluorescent beads added.


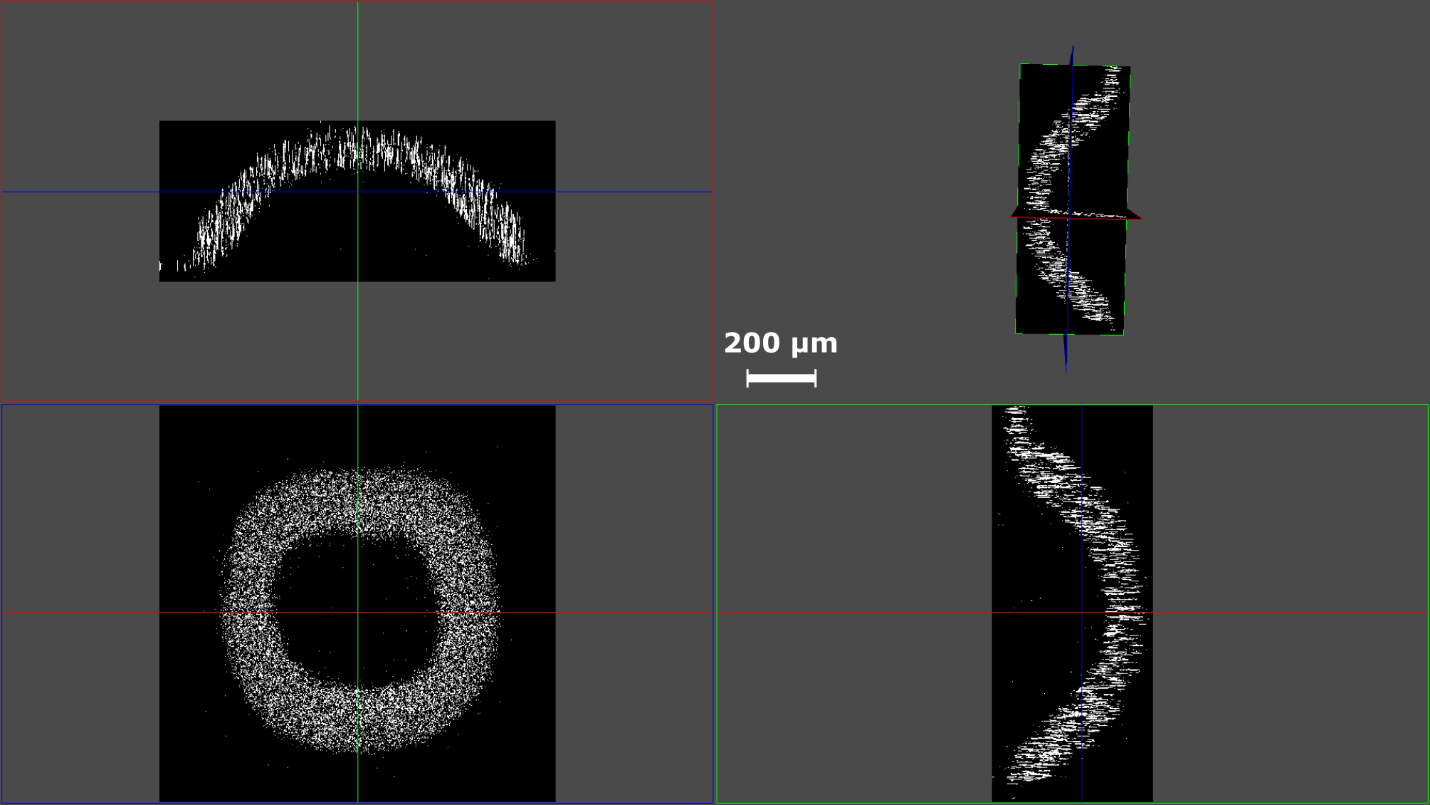


Figure S5. In the 3D viewing mode of confocal microscopy images, the deepest point of the hydrogel pocket was determined by scrolling through the z stacks in *zx* and *zy* direction.


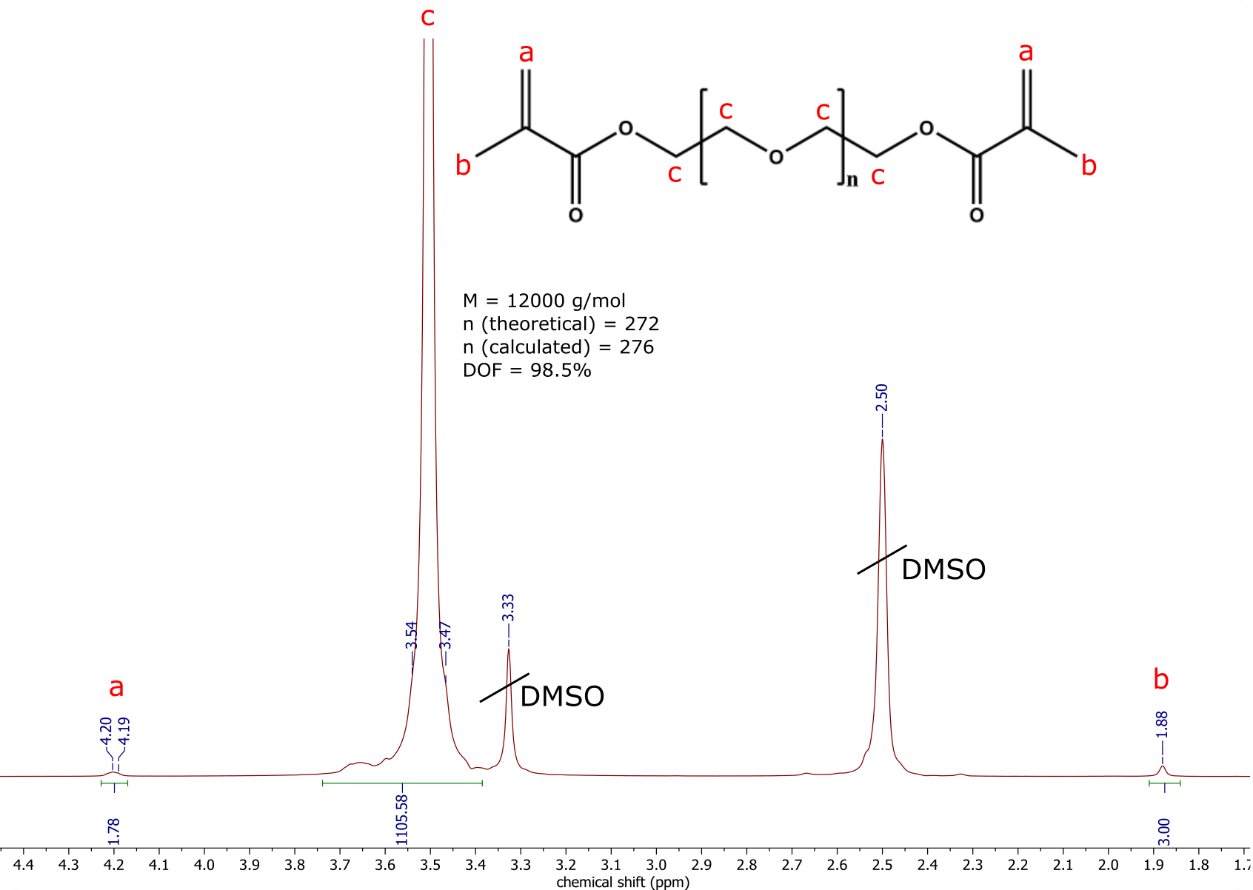


Figure S6. ^1^H-NMR spectrum (400 MHz, DMSO-d_6_) of PEG-dimethacrylate

(PEG(12000)-DMA).


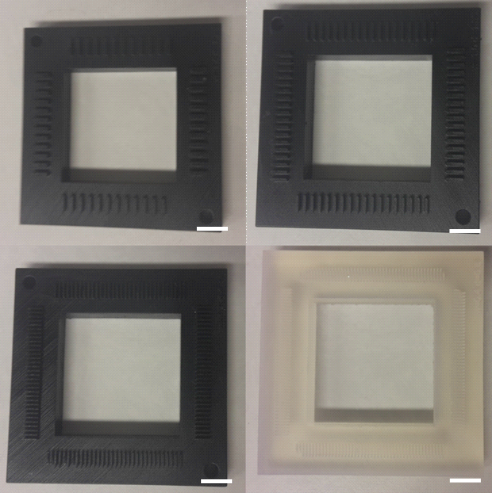


Figure S7. Photograph of four different 3D-printed frames used for PCL mesh formation. Thickness of the pegs and spacing between the pegs was varied between 600 μm - 2 mm, resulting in meshes with pore sizes from 200 µm - 1.8 mm. Scale bar = 1 cm.
